# Volumetric Growth of the Liver in the Human Fetus: An Anatomical, Hydrostatic, and Statistical Study

**DOI:** 10.1155/2015/858162

**Published:** 2015-08-27

**Authors:** Michał Szpinda, Monika Paruszewska-Achtel, Alina Woźniak, Celestyna Mila-Kierzenkowska, Gabriela Elminowska-Wenda, Małgorzata Dombek, Anna Szpinda, Mateusz Badura

**Affiliations:** ^1^Department of Normal Anatomy, Collegium Medicum of Nicolaus Copernicus University, Łukasiewicza 1 Street, 85-821 Bydgoszcz, Poland; ^2^Department of Medical Biology, Collegium Medicum of Nicolaus Copernicus University, Karlowicza 24 Street, 85-092 Bydgoszcz, Poland

## Abstract

Using anatomical, hydrostatic, and statistical methods, liver volumes were assessed in 69 human fetuses of both sexes aged 18–30 weeks. No sex differences were found. The median of liver volume achieved by hydrostatic measurements increased from 6.57 cm^3^ at 18–21 weeks through 14.36 cm^3^ at 22–25 weeks to 20.77 cm^3^ at 26–30 weeks, according to the following regression: *y* = −26.95 + 1.74 × age ± *Z*  × (−3.15 + 0.27 × age). The median of liver volume calculated indirectly according to the formula liver volume = 0.55 × liver length × liver transverse diameter × liver sagittal diameter increased from 12.41 cm^3^ at 18–21 weeks through 28.21 cm^3^ at 22–25 weeks to 49.69 cm^3^ at 26–30 weeks. There was a strong relationship (*r* = 0.91, *p* < 0.001) between the liver volumes achieved by hydrostatic (*x*) and indirect (*y*) methods, expressed by *y* = −0.05 + 2.16*x*  ± 7.26. The liver volume should be calculated as follows liver volume = 0.26 × liver length × liver transverse diameter × liver sagittal diameter. The age-specific liver volumes are of great relevance in the evaluation of the normal hepatic growth and the early diagnosis of fetal micro- and macrosomias.

## 1. Introduction

Since the fetal liver is a pivotal organ involved in fetoplacental metabolism, the assessment of liver volume is indispensable to satisfactory understanding of fetal physiology and the status of fetal growth and nutrition [1]. Aberrant fetal growth directly results in disparate alterations of fetal liver volume [2]. In pregnancy complicated by maternal insulin-dependent diabetes mellitus, the fetal liver volume accelerates by approximately 20% at every week of gestation when compared with normal controls [2]. In fetuses at 11 to 13 weeks with trisomy 21, the liver volume is considerably increased [3, 4]. On the other hand, significantly decreased liver volumes are typical of fetuses with growth restriction [5–8]. Determination of fetal liver volume* in utero* can mainly be achieved by preferable three-dimensional ultrasound, including either multiplanar or VOCAL (Virtual Organ Computer-Aided Analysis) techniques [3, 9], and sporadically by MRI, a costly method of low acceptability in pregnant women [10, 11]. To date, the liver volume of normal fetuses measured by 3D ultrasound has been found to increase disparately, from a linear function with age [12], through a log linear relationship [2] or a third-order polynomial regression with age [1] to an exponential model with crown-rump length [3] and with gestational age [13].

The current paper caps the morphometric investigation of the fetal liver, some outcomes of which concerning liver length and transverse and sagittal diameters have recently been published in the Surgical and Radiologic Anatomy [14]. To date, however, no nomograms have been computed by means of detailed direct measurements of liver volume in the human fetus. A limited number of articles focused on the liver volume accomplished indirectly by measurements of liver length and transverse and sagittal diameters in accordance with the two empirical formulae: liver volume = 0.45 × length × transverse diameter × sagittal diameter [15] or liver volume = 0.55 × length × transverse diameter × sagittal diameter [16]. In the light of the recently published INTERGROWTH-21st Project [17], in this study we aimed to concentrate on

- age-specific references for liver volume at varying gestational ages,
- possible sex differences in liver volume,
- the 3rd, 10th, 50th, 90th, and 97th smoothed centile curves for the liver volume over time (optimal growth curve),
- the relationship between liver volumes for the 50th centile measured directly and those calculated indirectly on the base of liver length and transverse and sagittal diameters.

## 2. Materials and Methods

The examinations were executed in the Department of Anatomy of the Ludwik Rydygier Collegium Medicum in Bydgoszcz. The current study was carried out on 69 autopsied formalin-fixed human fetuses of both sexes (32 male, 37 female) aged 18–30 (23.35 ± 3.39) weeks of Caucasian ethnic origin (Table 1), gathered in the years 1989–1999 from spontaneous miscarriages or stillbirths. First of all, fetuses from diabetic or manifold gravidities and fetuses affected by innate and chromosomal abnormalities or intrauterine growth restriction were omitted from the study. So, the sample included fetuses that could be considered as normal. Legitimate and moral dilemmas were granted by the Collegium Medicum Research Ethics Committee (KB 161/2013). According to the INTERGROWTH-21st Project, the fetal ages in weeks were precisely elaborated owing to the three subsequent criteria: (1) the fetal crown-rump length, (2) identified date of the start of the last motherly menstrual period, and (3) a combination of known values of the five fetal anthropometric measurements: head circumference, biparietal diameter, occipitofrontal diameter, abdominal circumference, and femur length assessed by early second-trimester ultrasound scan (ultrasound age) [14, 17]. The crown-rump length was measured with the use of a flexible caliper from the top of the head (crown) to the bottom of the buttocks (rump) of the fetus in its natural C-shaped position [18].

### 2.1. Anatomical Method

After having been immersed for 12–24 months in 10% neutral buffered formalin solution, the fetuses were subjected to anatomical dissection by both median and transverse laparotomies under 10-fold magnification with the use of a stereoscope with Huygens ocular. By cutting off peritoneal ligaments, diaphragm, inferior vena cava, and structures at the porta hepatis, the liver was freed and removed out of the abdominal cavity.

### 2.2. Hydrostatic Method

**Figure 1:**
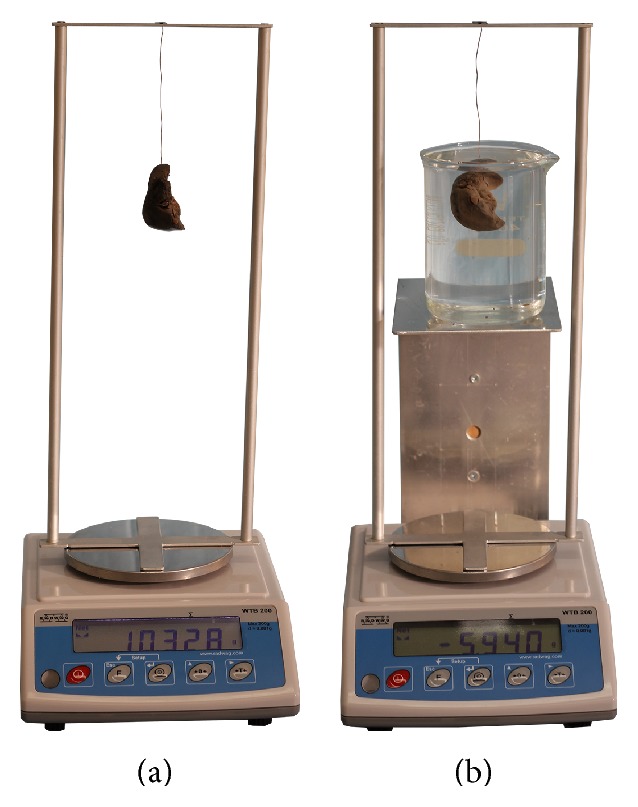
A double weighing procedure to obtain the weight of the liver in air (a) and distillate water (b).

Subsequently, every isolated liver as an object of multifaceted form was subjected to direct volumetric analysis, with the use of a hydrostatic method, grounded in Archimedes' principle [19]. Therefore, the liver submerged in water loses weight quantitatively tantamount to the weight of the water displaced by the liver. Consequently, a dual weighing method (Figure 1) was then used to acquire the weight (in g) of the liver in air (*W*_*A*_) and in distillate water (*W*_*W*_), taking into account the specific gravity (in g/cm^3^) of water (*G*_*W*_) and air (*G*_*A*_) in the range of temperature between 14 and 20°C [19]. Thus, for every fetus, the liver volume in cm^3^ (*V*) was accurately calculated by the succeeding formula: *V* = *W*_*A*_ − *W*_*W*_/*G*_*W*_ − *G*_*A*_. Of note, notwithstanding that both *W*_*A*_ and *W*_*W*_ are considerably influenced by formalin fixation, the difference between these two expressed in the nominator (*W*_*A*_ − *W*_*W*_) is utterly unfettered by the weight gain of formalin-fixed structures. Furthermore, we calculated the liver volume, extrapolated through a series of indirect, previously achieved measurements [14], according to the following formula: liver volume = 0.55 × liver length × liver transverse diameter × liver sagittal diameter.

### 2.3. Statistical Analysis

In an unceasing attempt at minimizing measurement and observer bias, all the measurements were performed by one investigator (Monika Paruszewska-Achtel). Each measurement was executed three times (*V*_1_, *V*_2_, *V*_3_) under the same circumstances but at different times, and the average was involved in individual numerical data. In the current study, the statistical program* Statistica 10* was used. The intraobserver variation between the reiterated measurements was evaluated by ANOVA for repeated measurements and post hoc RIR Tukey test. The numerical data were verified for normality of distribution (Shapiro-Wilk's test) and for homogeneity of variance (Levene's test). As the first step of the statistical analysis, the Mann-Whitney *U* test for unpaired variables was preferred to evaluate the likelihood of appearance of statistically significant differences in values with relation to sex. Since the fetuses studied were collected into 12 one-week intervals inadequately dispersed with fetal age, the first four intervals (18–21 weeks), the consecutive four intervals (22–25 weeks), and the last four intervals (26–30 weeks) were aggregated. At first, we tested sex differences between the three forenamed age groups, 18–21 (*n* = 24), 22–25 (*n* = 27), and 26–30 (*n* = 18) weeks, and later for the whole sample. Having considered the sample size of the groups, the Kruskal Wallis test for unpaired data proved to be more appropriate for comparisons to check whether significant differences in liver volume occurred with fetal age. The algebraic volumetric data were correlated to fetal age, and linear and nonlinear regression analysis were used to achieve the specific best-fit growth curve for liver volume against fetal age. The creation of charts of the liver volume followed the Altman-Chitty method [14, 18]. In such a way, we established the mean, standard deviation, and the five centiles (3rd, 10th, 50th, 90th, and 97th) for liver volume at each gestational age. After that, the 3rd, 10th, 50th, 90th, and 97th smoothed centile curves for the liver volume* versus* gestational age were computed. Linear regression analysis was used to examine the relationship between the liver volumes obtained directly and indirectly. Typically, statistically significant differences were deliberated at *p* < 0.05.

## 3. Results

No statistically significant difference (*p* = 0.291) was found in evaluating intraobserver reproducibility of three liver volume measurements that in the fetuses aged 18–30 weeks averaged *V*_1_ = 14.20 ± 7.09 cm^3^, *V*_2_ = 14.00 ± 6.86 cm^3^, and *V*_3_ = 14.01 ± 6.91 cm^3^, respectively. Since no significant sex difference was observed in liver volume (Table 2), no attempt was made to further separate the results obtained according to males and females. Therefore, both its direct measurements and its indirect calculations have been collectively summarized for both sexes in Table 3. Obviously, having previously been published, the three aforementioned morphometric parameters of the liver [14], namely, its length and transverse and sagittal diameters, were excluded from Table 3. On the contrary, a statistically significant increase in liver volume was found in fetuses aged 18–21, 22–25, and 26–30 weeks.

**Figure 2:**
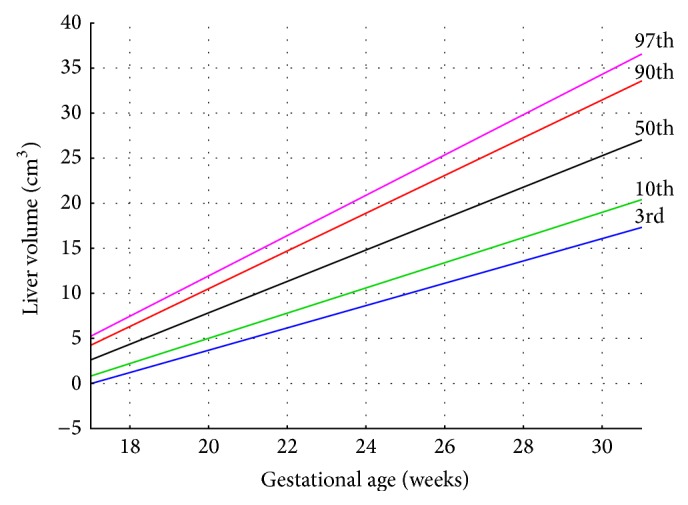
The 3rd, 10th, 50th, 90th, and 97th smoothed centiles for liver volume* versus* gestational age.

The median value of liver volume achieved by direct hydrostatic measurements was found to increase from 6.57 cm^3^ at the age of 18–21 weeks through 14.36 cm^3^ in fetuses aged 22–25 weeks to 20.77 cm^3^ at 26–30 weeks of gestation. The best suitable curves for the liver volume were presented in the following five cutoff points: 3rd, 10th, 50th, 90th, and 97th centiles (Figure 2). The two corresponding formulae for the estimation of the mean and SD (in cm^3^) of liver volume in accordance with gestational age (in weeks) were displayed as follows: “−26.95 + 1.74 × age” and “−3.15 + 0.27 × age,” respectively. The specific centiles were calculated as “mean ± *Z* × SD.” From a statistical point of view, the value of *Z* depends on a particular centile and constantly equals −1.88 for the 3rd centile, −1.28 for the 10th centile, 0 for the 50th centile, +1.28 for the 90th centile, and +1.88 for the 97th centile [17]. Thus, the values of liver volume for particular centiles in relation to gestational age in weeks were calculated by the following linear regressions: *y* = −26.95 + 1.74 × age ± *Z* × (−3.15 + 0.27 × age). It is noteworthy that the whole model (statistics *F*) and its parameters were statistically significant (*p* < 0.001). According to this formula, for the 50th centile (*Z* equals 0), the fetal liver volume grew proportionately during the study period at a rate of 1.74 cm^3^ per week. Of note, the coefficient of determination (*r* ^2^) for the 50th centile reached the value of 0.79.

**Figure 3:**
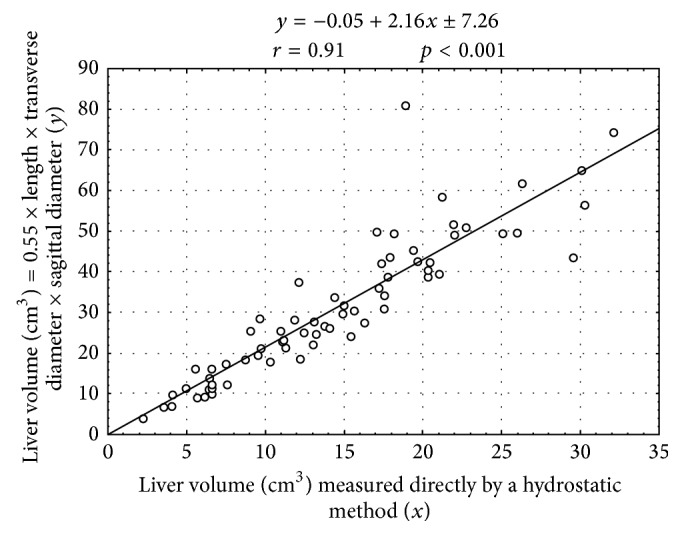
Linear relationship between the liver volumes for the 50th centile achieved by the two methods.

On the other hand, during the study period, the median value of liver volume (equivalent to the 50th centile) calculated indirectly according to the formula liver volume = 0.55 × liver length × liver transverse diameter × liver sagittal diameter increased from 12.41 cm^3^ at 18–21 weeks through 28.21 cm^3^ at 22–25 weeks to 49.69 cm^3^ at 26–30 weeks. The calculated liver volume considerably predominated over the measured 50-centile liver volume. The measured-to-calculated liver volume ratio for the 50th centile attained the value of 0.48 ± 0.08 throughout the analyzed period. There was a strong relationship (*r* = 0.91, *p* < 0.001) between the liver volumes for the 50th centile (Figure 3), achieved by hydrostatic (*x*) and indirect (*y*) methods, expressed by the following linear function: *y* = −0.05 + 2.16*x* ± 7.26, where “±7.26” meant the standard error of the estimate. In order to obtain equal values of liver volume in both methods, a constant of 0.55 should be substituted with 0.26 (in brief, 0.55 × 0.48 = 0.26).

## 4. Discussion

The present study is no veracious representation of growth in itself but comprises a cross-sectional design of the longitudinal growth of liver volume supported by the numerical evidence obtained from a relatively numerous sample (*n* = 69) of normal autopsied formalin-fixed fetuses aged 18–30 weeks. Of note, the 12–24-month formalin preservation substantially alters the weight and density of fixed organs. The weight gain of formalin-fixed organs may even fluctuate from 10–12% for the encephalon to 20–25% for the heart and liver, when compared to their initial weight [20]. As a consequence of tissue shrinkage, formalin preservation may additionally influence volume of organs in question, predominantly with relation to isolated organs [14, 20]. On the contrary, with relation to organs preserved* in situ* in the sealed abdominal cavity, formalin fixation influences little (0.5–1.0%) their volumes [18, 19]. This has been supported by the fact that the liver length and transverse and sagittal diameters in the material under examination are harmonious with those in* in utero *fetuses of the same age assessed by 3D ultrasound [14]. Therefore, from the clinical perspective, visceral measurements and growth curves obtained anatomically are comparable with particular ultrasonic measurements [14, 21]. As stated by Breeze et al. [21], conventional autopsy still remains the gold reference standard in the quantitative evaluation of fetal organs. As a result, the findings obtained in this study can both aggregately be discussed and straightly be adapted* in vivo* to the fetus.

The precise estimation of fetal ages in the material under examination has been compatible with the Fetal Growth Longitudinal Study, part of the International Fetal and Newborn Growth Consortium for the 21st Century (INTERGROWTH-21st) Project [17]. In this study, the fetuses could not suffer from intrauterine growth retardation since their gestational, amenorrhea, and ultrasound ages proved to be highly (*r* = 0.99, *p* < 0.001) correlated [14]. It should be emphasized that we performed liver volume determination by a direct and clearly precise method based on Archimedes' principle [19]. Since both the material studied and the method used have been apposite, our findings can be considered factual.

In the material under examination, we found no statistically significant male-female differences in liver volume. These findings are in line with previous reports in which anatomical [22], 3D ultrasound [7, 16, 23], and MRI [11, 21, 24] methods were used.

The anatomical research by Albay et al. [22] found the liver volume to grow from 18.0 ± 0.6 cm^3^ in the first trimester through 14.8 ± 12.5 cm^3^ in the second trimester and 45.7 ± 21.0 cm^3^ in the third trimester to 71.8 ± 19.9 cm^3^ in full term fetuses. Guihard-Costa et al. [20] evaluated liver weight in 640 autopsied formalin-fixed human fetuses aged 13–42 weeks. The liver weight increased from 3.09 ± 0.27 g in fetuses aged 12-13 weeks to 161.94 ± 37.78 g in fetuses aged 41-42 weeks. It is noteworthy that for the 5th and 95th centiles the liver volume averaged 2.64 cm^3^ and 3.54 cm^3^ at 13-14 weeks and 99.80 cm^3^ and 224.08 cm^3^ at 41-42 weeks of gestation. Since the density of the fetal liver did not change throughout the gestation, the weight growth dynamics of the fetal liver precisely revealed its volumetric growth [8]. This was also confirmed by Breeze et al. [21], who reported both liver volume (*y*) and liver weight (*x*) to increase parabolically, with a reciprocal relationship, best modelled by the following linear function: *y* = 2.93 + 0.87*x*. In the material under examination, the median value of liver volume achieved by direct hydrostatic measurements grew from 6.57 cm^3^ at 18–21 weeks through 14.36 cm^3^ at 22–25 weeks to 20.77 cm^3^ at 26–30 weeks.

For the growing fetal liver, we tested three regression models, namely, third-degree polynomial, natural logarithmic, and linear functions. The choice of the best-fit model encountered the following criteria: the greatest *r* ^2^ value, all coefficients different from 0, and the lowest SD of regression. Regrettably, in the estimated third-degree polynomial model, its parameters proved to be statistically insignificant (*p* = 0.254). The linear and logarithmic models displayed approximated *r* ^2^ values: 0.794 and 0.791, respectively. However, the linear model was characterized by the lowest values of both standard deviation for parameters and the standard error of the estimate for the whole model. Of note, residual value analysis showed normality of distribution for both linear and logarithmic models. In the linear and logarithmic models, there were three and four extremal values, respectively, for which standardized residuals were beyond the range of (−2, +2). Finally, the linear model was of best-fit for our empirical data throughout the analyzed fetal period. In this study, the algebraic data have been presented in an analogous manner as the INTERGROWTH-21st Project data [17], comprising the fitted 3rd, 10th, 50th, 90th, and 97th smoothed centile curves. Therefore, the best-fit growth dynamics was expressed by the linear function *y* = −26.95 + 1.74 × age ± *Z* × (−3.15 + 0.27 × age). According to such a growth pattern for the 50th centile (*Z* = 0), the rate of hepatic volumetric growth averaged 1.74 cm^3^ per week.

With the use of 2D ultrasound, Gimondo et al. [15] indirectly estimated liver volume by measuring the length and transverse and sagittal diameters of the fetal liver and multiplying them by a constant of 0.42. Chang et al. [16] tested hypothesis whether the liver volume obtained by 2D ultrasound from Gimondo's formula [15], that is, liver volume = 0.45 × length × transverse diameter × sagittal diameter, is tantamount to that determined directly by 3D ultrasound. As it turned out, a volume constant of 0.42 tenuously reflected direct volumetric determination because of its underestimation of the fetal liver volume. Therefore, the new reference volume constant of 0.55 was substituted for the old one to obtain the formula: liver volume = 0.55 × liver length × liver transverse diameter × liver sagittal diameter, of practical meaning if only 2D ultrasound is available. The new formula yielded a more accurate estimation of liver volume, because it proved to be closer to and displayed no difference when compared to the actual liver volume evaluated with 3D ultrasound [16].

The introduction of 3D ultrasound has considerably enhanced diagnostic power in maternal fetal medicine. This method allows determination of hepatic volume by slicing through collected images and recording a truncated pyramidal volume [5]. The superior outline of the liver referring to the diaphragm is easy to delineate, while its inferior outline fades away. Volume determination is possible in only technically satisfactory ultrasonic liver recordings by multiplanar stepping through the liver and then by building the total liver volume equal to the sum of all individual volumes of parallel slices as follows [5]. Firstly, a reference plane (mostly a frontal cross section of the liver, prior to the stomach) has to be selected and fixed as an anchor. Secondly, in a concurrently obtainable sagittal cross section, the contour of the liver is manually traced and the liver surface area is measured slice by slice in some 10 sagittal projections flanked by the most lateral left and right points of the diaphragm in the frontal plane. Thirdly, the system integrates and calculates the total liver volume automatically. Chang et al. [16] showed that with respect to fetal liver volume 3D ultrasound is superior to 2D ultrasound and should be used for reaching its accurate determination. Ioannou et al. [25] identified six 3D ultrasound studies reporting normal volumes of the fetal liver at 32 weeks of gestation [1, 5, 8, 12, 16, 25]. However, because of poor standardization of volumetric methodology, there were wide discrepancies, even by 30% in reported normal hepatic volumes. The reference group was considered the most numerous one (*n* = 226) presented by Chang et al. [1] with the liver volume of 54.57 cm^3^ that turned out to be the least of all. The remaining liver volumes in the 32-week fetus averaged 62.02 cm^3^ by Chang et al. [16], 63.3 cm^3^ by Kuno et al. [8], 66.42 cm^3^ by Boito et al. [5], 72.02 cm^3^ by Laudy et al. [12], and 74 cm^3^ by Rizzi et al. [26]. Chang et al. [16] demonstrated the liver volumetric growth in 55 fetuses aged 20–31 weeks that followed proportionately: *y* = −78.29 + 4.38 × age  (*r* = 0.85, *p* < 0.001). In another study by Chang et al. [1] carried out on 226 fetuses at the age of 20–40 weeks, the liver volume increased from 11.73 ± 1.39 to 131.59 ± 16.71 cm^3^, in accordance with the following cubic function: *y* = −398.26 + 46.20 × age − 1.76 × (age)^2^ + 0.02 × (age)^3^  (*r* = 0.97, *p* < 0.001). Furthermore, these authors presented differentiated regression lines of fetal liver volume with relations to biparietal and occipitofrontal diameters, head and abdominal circumferences, femur length, and estimated fetal weight. The linear relationship (*y* = 0.04*x* − 7.52; *r* = 0.93, *p* < 0.001) between liver volume and estimated fetal weight was noted. The second-degree polynomial growth of liver volume was found in relation to both occipitofrontal diameter (*y* = 3.23*x* ^2^ − 40.98*x* + 146.5; *r* = 0.85, *p* < 0.001) and abdominal circumference (*y* = 0.24*x* ^2^ − 6.53*x* + 60.05; *r* = 0.93, *p* < 0.001). The third-degree polynomial growth in liver volume occurred relative to biparietal diameter (*y* = 1.74*x* ^3^ − 31.38*x* ^2^ + 195.8*x* − 396.53; *r* = 0.90, *p* < 0.001), head circumference (*y* = 0.04*x* ^3^ − 2.80*x* ^2^ + 61.81*x* − 444.77; *r* = 0.90, *p* < 0.001), and femur length (*y* = 2.50*x* ^3^ − 30.99*x* ^2^ + 138.22*x* − 195.1; *r* = 0.92, *p* < 0.001). Laudy et al. [12] found the liver volume to increase proportionately in 25 fetuses, including small-, appropriate-, and large-for-gestational-age subjects. Because the fetuses did not constitute a homogenous sample, such a growth pattern could be doubtful for estimating the growth of liver volume during normal pregnancy. Kuno et al. [8] measured liver volume every 2 weeks in 14 appropriate-for-gestational-age fetuses from 20 weeks of gestation until delivery. The growth dynamics for liver volume was found to follow curvilinearly (parabolically). According to Boito et al. [2], after a logarithmic transformation of volumes in cm^3^, the log_10_ linear regression for fetal liver volume against gestational age in weeks was calculated as follows: log_10_ liver volume = 0.14 × age − 0.31 in the normal group. Gielchinsky et al. [3] used an improved 3D ultrasound method, that is, the VOCAL technique for measuring liver volumes. This method allows liver volume determination by rotating the organ around a fixed axis through a number of sequential steps [10]. These authors found that the liver volume of 200 normal fetuses aged 11–13 weeks as a function of crown-rump length (CRL) grew exponentially from 0.5 cm^3^ at 11 weeks (CRL 45 mm) to 2.5 cm^3^ at 13 weeks (CRL 84 mm) according to the following formula: log_10_ liver volume = −1.20 + 0.02 × CRL (*r* ^2^ = 0.86, *p* < 0.001). This was consistent with the autopsy findings by Archie et al. [13], who confirmed that liver weight grew exponentially with gestation from 1 g at 12 weeks through 5 g at 16 weeks to 30 g at 30 weeks.

In the material under examination, we compared the liver volume directly achieved by a hydrostatic method based on Archimedes' patent with the liver volume indirectly calculated due to the following formula: liver volume = 0.55 × liver length × liver transverse diameter × liver sagittal diameter, using the three aforementioned numerical parameters of the fetal liver recently published by us [14]. In doing so, the median value of liver volume calculated indirectly in accordance with the formula liver volume = 0.55 × liver length × liver transverse diameter × liver sagittal diameter revealed an increase from 12.41 cm^3^ at 18–21 weeks through 28.21 cm^3^ at 22–25 weeks to 49.69 cm^3^ at 26–30 weeks of gestation. Independently of fetal age, the calculated liver volume substantially predominated over the measured liver volume. We confirmed a strong relationship (*r* = 0.91, *p* < 0.001) between the liver volumes obtained by hydrostatic (*x*) and indirect (*y*) methods, expressed by the linear model: *y* = −0.05 + 2.16*x* ± 7.26. The measured-to-calculated liver volume ratio attained the value of 0.48 ± 0.08 throughout the analyzed period. However, our volumetric comparisons have accentuated that the calculation of fetal liver volume essentially overestimates the results obtained by a hydrostatic method. Because calculated liver volumes are well-suited with those achieved by 3D ultrasonic measurements [1, 16], we opine that liver volume determination provided by 3D ultrasonography does not reveal factual results. Supportive evidence for this concept is the finding that in the material under examination the liver length and transverse and sagittal diameters were harmonious with those assessed by 3D ultrasound in* in utero* fetuses matched for gestational age [14]. According to our calculations, the best-fit constant should be 0.26 in the following formula: liver volume = 0.26 × liver length × liver transverse diameter × liver sagittal diameter.

Having discussed the quantitative growth of fetal liver volume, we would like to highlight some germaneness of liver volume determination in the fetus. Owing to the best-fit growth models for the mean and SD for liver volume, readers can readily calculate any chosen centiles according to gestational age. It is essential to know the value of *Z* that constantly equals −1.88 for the 3rd centile, −1.28 for the 10th centile, 0 for the 50th centile, +1.28 for the 90th centile, and +1.88 for the 97th centile [17]. As reported by Boito et al. [2], liver volume was found to be greater by 20% in 32 fetuses of diabetic women (45.9 ± 34.0 cm^3^) when compared to 32 normal controls (38.3 ± 28.7 cm^3^) at the age of 18–36 weeks of gestation. The log_10_ linear regression for fetal liver volume against gestational age in weeks was expressed by the following relationship: log_10_ liver volume = 0.14 × age − 0.04 in the diabetic group. Furthermore, liver volume was positively related to maternal glycosylated hemoglobin concentrations (HbA1c), in accordance with the following regression: log_10_ liver volume = 0.14 × age + 0.08 × HbA1c − 0.48. This means that the liver volume was increased by 8% for each unit increase in maternal HbA1c and by 14% per week of gestational age. Astonishingly enough, using the VOCAL technique, Dubé et al. [9] exposed no difference in fetal liver volume during the third trimester in 10 women with normal glucose tolerance and in 17 women with gestational diabetes mellitus. In the diabetic group, the fetal liver volume increased from 52 ± 24 cm^3^ at 24–28 weeks through 90 ± 16 cm^3^ at 32 weeks to 124 ± 23 cm^3^ at 36 weeks of gestation. The fetal liver volumes in the control group were characterized by the following values: 54 ± 16 cm^3^, 89 ± 17 cm^3^, and 128 ± 31 cm^3^, respectively. Moreover, differences in liver volume between 28 and 32 weeks (35 ± 17 cm^3^ *versus* 36 ± 26 cm^3^), 32 and 36 weeks (39 ± 25 cm^3^ *versus* 29 ± 22 cm^3^), and 28 and 36 weeks (75 ± 37 cm^3^ *versus* 70 ± 35 cm^3^) proved to be statistically insignificant. Of note, as reported by Archie et al. [13] in 73% of fetuses with trisomy 21, the liver was enlarged over the 95th percentile, as a consequence of the disturbed hematopoiesis with intrahepatic expansion of the “leukemia-initiating” progenitor population. On the contrary, in fetal growth restriction, reduction is more expressed for hepatic volume than for head or upper abdominal circumference [5]. According to Kuno et al. [8], in 10 small-for-gestational-age fetuses from 20 weeks of gestation until delivery, liver volume was decreased and followed in accordance with the following formula: liver volume = 167 − 14.6 × menstrual age + 0.36 × menstrual age (*r* ^2^ = 0.88, *p* < 0.001). Due to the brain-sparing effect in the small-for-gestational-age fetus, a decrease in liver volume is much more conspicuous than that in brain weight, and the former may thus contribute to the early recognition of fetal growth restriction [5, 23, 27].

To the best of our knowledge, this research paper is the first autopsy study to endow us with direct hepatic volume measurements. Consequently, our results are not affected by many ultrasound disadvantages attributable to the difficulty in outlining the whole contour of the liver because of inherent ultrasonic artifacts (image speckle, signal attenuation, and acoustic shadowing), unclear reference planes and anatomical landmarks, heterogeneity of 3D ultrasound system platforms and methods, fetal movement artifacts, the fetal back in the anterior position, and reduced amniotic fluid volume [5, 25]. It is noteworthy that, in 3D volumetry, errors in caliper placement will be multiplied over the volume [25]. The main limitations of this study appear to be (1) a lack of fetuses younger than 18 weeks and older than 30 weeks of gestation, (2) retrospective analysis without prospective ultrasound quality control, (3) measurements conducted by a single observer in a blind fashion, and (4) a lack of interobserver variability.

In summary, both the numerical data and computed nomograms for liver volume obtained in this study improve our information of hepatic quantitative anatomy in human fetuses. This may serve as a suitable reference in monitoring normal fetal development and screening for disturbances in fetal growth.

## 5. Conclusions

The fetal liver volume does not reveal sex differences. The growth of fetal liver volume follows a linear function. The regression equations for the estimation of the mean and standard deviation of liver volume allow for calculating any desired centiles according to gestational age. 3D ultrasound techniques considerably overestimate liver volumes relative to an accurate hydrostatic method. The liver volume should be calculated by the following formula: liver volume = 0.26 × liver length × liver transverse diameter × liver sagittal diameter. The age-specific references for liver volume at varying gestational ages are of great relevance in the evaluation of the normal hepatic growth and the early diagnosis of fetal micro- and macrosomias.

## Figures and Tables

**Table 1:** Distribution of the fetuses examined.

Fetal age [weeks]	Crown-rump length [mm]			*n*	Sex	
	Median	Minimum	Maximum		Males	Females
18	139.5	131.0	143.0	4	3	1
19	152.5	145.0	155.0	6	4	2
20	161.0	159.0	167.0	7	3	4
21	175.0	170.0	180.0	7	5	2
22	185.5	181.0	190.0	6	1	5
23	199.5	195.0	204.0	6	4	2
24	212.0	205.0	214.0	10	2	8
25	215.0	215.0	220.0	5	2	3
26	233.0	225.0	233.0	3	1	2
27	240.5	235.0	242.0	4	2	2
28	253.0	247.0	253.0	7	1	6
30	264.0	263.0	265.0	4	4	0

Note: for anatomists dealing with fetuses, the most objective information for establishing fetal ages is the crown-rump length, when compared to the known data of the beginning of the last maternal menstrual period or to ultrasonic measurements of head circumference, biparietal diameter, occipitofrontal diameter, abdominal circumference, and femur length.

**Table 2:** Liver volumes in both sexes measured directly by a hydrostatic method.

Fetal age [weeks]	*n*	Liver volume [cm^3^]						*p* value
		Males			Females			
		Median	Minimum	Maximum	Median	Minimum	Maximum	
18–21	24	6.46	2.23	12.14	10.31	4.94	13.24	0.128
22–25	27	15.41	6.58	25.93	14.21	7.46	26.30	0.758
26–30	18	21.48	17.36	32.10	20.77	14.95	29.54	0.477
18–30	69	11.60	2.23	32.10	14.36	4.94	29.54	0.166

**Table 3:** Liver volumes measured directly by a hydrostatic method and calculated indirectly through a series of indirect, previously achieved measurements, according to the following formula: liver volume = 0.55 × length × transverse diameter × sagittal diameter.

Liver volume [cm^3^]							
Fetal age [weeks]	*n*	Measured directly by a hydrostatic method based on Archimedes' patent			Calculated indirectly according to the following formula: liver volume = 0.55 × length × transverse diameter × sagittal diameter		
		Median	Minimum	Maximum	Median	Minimum	Maximum
18–21	24	6.57^(1)^	2.23	13.24	12.41^(a)^	4.02	28.51
22–25	27	14.36^(2)^	6.58	26.30	28.21^(b)^	16.17	49.60
26–30	18	20.77^(3)^	14.95	32.10	49.69^(c)^	31.68	80.96

Note: liver volumes measured directly differ significantly in columns as follows: for (1) versus (2), p < 0.001; for (1) versus (3), p < 0.001; and for (2) versus (3), p = 0.007.

Liver volumes calculated indirectly differ significantly in columns as follows: for (a) versus (b), p < 0.001; for (a) versus (c), p < 0.001; and for (b) versus (c), p = 0.003.

Liver volumes measured directly and calculated indirectly differ significantly in rows: for (1) versus (a), (2) versus (b), and (3) versus (c), p < 0.001.
